# Phylogenetic Insights into the Evolutionary History of the *RSPO* Gene Family in Metazoa

**DOI:** 10.3390/genes16050477

**Published:** 2025-04-23

**Authors:** Jia Cheng, Ling Yang, Shiping Wang, Kaiyong Luo, Senlin Luo, Yang Dong, Ya Ning, Weibin Wang

**Affiliations:** 1 College of Food Science and Technology, Yunnan Agricultural University, Kunming 650201, China; chenj@dongyang-lab.org (J.C.); lky@dongyang-lab.org (K.L.); lsl@dongyang-lab.org (S.L.); 2 Yunnan Provincial Key Laboratory of Biological Big Data, Yunnan Agricultural University, Kunming 650201, China; jxaa174751@163.com; 3 Institute of Agro-Products of Processing and Design, Hainan Academy of Agricultural Sciences, Haikou 571100, China; yangl@dongyang-lab.org; 4 College of Science, Yunnan Agricultural University, Kunming 650201, China; ningya0428@126.com

**Keywords:** RSPO gene family, molecular evolution, phylogenetic analysis, the enhancer of Wnt signaling

## Abstract

**Background**: The *RSPO* gene family encodes secreted glycoproteins that are rich in cysteine, which generally serve as activators of the Wnt signaling pathway in animals. Four types of this family have been identified in a few model species. However, the evolution of the family remains unclear. **Methods**: In this study, we identified a total of 1496 RSPO homologs through an extensive survey of the *RSPO* genes in 430 animals. Gene family clustering and phylogenetic analysis identified four major subtypes of the family (RSPO1–RSPO4) and clarified their distribution of copy number in different species. **Results and Conclusions**: Members of the RSPO4 subfamily that were closest to ancestral forms existed in both Deuterostomes and Protostomates, and we speculate that representatives of this subfamily already existed in *Urbilatera*, the last common ancestor of Deuterostomes. Particularly, in some RSPO3 subtypes of Actinopterygii (ray-finned fishes), an FU repeated motif with three conserved cysteines was identified. Further conservative analysis of amino acids and alignment of tertiary protein structure revealed the potential functional sites for each subgroup. The results provide insight into the phylogenetic relationships and evolutionary patterns of conserved motifs of *RSPO* family genes in animal kingdoms, which will guide further studies on the biological functions of RSPO in other non-model species.

## 1. Introduction

Cells, as the fundamental units of life, communicate through secreted signals, leading to the evolution of complex signaling networks in multicellular organisms. Among these, the Wnt signaling pathway is highly conserved and plays a crucial role in development, cellular differentiation, and tissue homeostasis [1,2,3,4,5,6]. The Wnt signaling is transmitted through three major pathways: the canonical Wnt (Wnt/β-catenin), the noncanonical Wnt/Ca^2+^, and the noncanonical planar cell polarity (PCP) [7,8,9]. Among them, the canonical Wnt/β-catenin signaling pathway is the most extensively studied and plays a critical role in regulating cell proliferation, differentiation, maturation, and tissue development [6]. The pathway is activated upon binding of extracellular Wnt ligands to LRP5/6 and frizzled (FZD) membrane receptors, leading to dissociation of β-catenin from the degradation complex, β-catenin stabilization and nuclear translocation, and subsequent transcriptional regulation of target genes in the nucleus. To maintain cellular homeostasis and prevent overactivation of signaling, the pathway is tightly regulated at multiple levels [10]. The intracellular APC containing destruction complexes inhibit the pathway by inducing degradation of β-linker proteins and consequently inhibiting the pathway [11]. Another level of negative regulation is provided by ZNRF3 and RNF43 ubiquitin ligases, which reduce membrane Wnt receptor availability and subsequent downstream β-cyclin signaling by promoting degradation of LRP5/6 and FZD receptors [12,13].

Recent studies have identified R-spondin (RSPO) proteins as significant amplifiers of the canonical Wnt/β-catenin signaling pathway [14]. Binding of RSPO to LGR receptors and RNF43/ZNRF3 leads to the internalization of the RSPO-LGR-RNF43/ZNRF3 complex, thereby enhancing Wnt signaling [15,16,17,18,19]. In the absence of RSPO, the E3 ubiquitin ligases RNF43 and ZNRF3 act as negative regulators by mediating the ubiquitination and subsequent degradation of Wnt receptors FZD and LRP [20]. RSPO can also enhance the Wnt/β-catenin signaling pathway independently of ZNRF3/RNF43 or LGR binding [21,22,23]. Furthermore, in addition to classical Wnt signaling, RSPO has been found to regulate non-classical Wnt/PCP signaling by binding to HSPG syndecan-4 and LGR4,5, thereby regulating protozoal embryonic gastrulation and head cartilage morphogenesis [17,24]. Beyond enhancing WNT signaling, RSPO proteins have been found to antagonize bone morphogenetic protein (BMP) signaling through ZNRF3-mediated downregulation of BMP receptors [21,22,25,26,27]. The *RSPO* gene family comprises four members—RSPO1, RSPO2, RSPO3, and RSPO4—each sharing conserved structural domains. These domains include an N-terminal signal peptide, two adjacent furin-type cysteine-like domains (FU-CR1 and FU-CR2), a conserved thrombospondin type 1 repeat (TSR-1), and a variable C-terminal region consisting of a positively charged basic amino acid (BR) [20]. The first *RSPO* gene family member, hPWTSR (later renamed hRSPO3), was identified in 2002 through high-throughput sequencing of a human embryonic brain cDNA library [28]. In 2004, mouse *Rspo1* was discovered in the neural tube roof plate and named “Roof plate specific-Spondin (R-spondin)” [29]. The identification of RSPO2 and RSPO4 followed [14,20]. Studies have shown that the furin-like domains are essential for RSPO proteins to activate the Wnt signaling pathway [30,31,32], while the thrombospondin type 1 (TSP1) domain is involved in vascular physiology and disease processes [33,34,35]. Structural simulations of human RSPO4 suggest that its TSP1 domain contains a positively charged groove, enhancing its ability to bind cell surface heparin [36].

Wnt signaling is frequently activated in various cancers, with RSPOs functioning as positive regulators of the Wnt pathway. Numerous studies have demonstrated that *RSPO* family members are closely associated with cancer development. For example, it was discovered that the expression of *RSPO2* and *RSPO3* was upregulated in some colorectal cancers (CRCs) and breast malignancies and breast cancers, whereas overexpression of *RSPOs* in both cases was related to an activated Wnt signaling gene expression profile [37]. *RSPO2* overexpression leads to tumor formation in the mouse liver in a Hippo/Yap-dependent manner [38]. *RSPO3* exhibited a tumor-promoting effect in bladder cancer cells through activation of Wnt/β-catenin and Hedgehog signaling pathways [39]. Amplification of *RSPO1* signaling induces granulosa cell fate defects and cancers in mouse adult ovary [40]. *RSPO4* overexpression facilitates tumor cell invasion and migration [41]. In addition to their roles in cancer progression, *RSPO* family members are also critically involved in a wide range of developmental processes across animal species. *RSPO1* mutations can cause female-to-male XX sex reversal and are critical for germ cell development [42,43,44]. RSPO2 plays key roles in limb, respiratory tract, and craniofacial development and contributes to tendon/ligament homeostasis and repair [32,45,46,47,48]. Disruption of the RSPO3 leads to severe vascular defects, especially in the placenta [49,50]. Mutations in the gene encoding the RSPO4 cause autosomal recessive anonychia [51].

The Wnt signaling pathway is significantly conserved throughout metazoan lineages [52,53,54,55]. The *RSPO* gene family, as an important secreted ligand in this signaling pathway, exhibits evolutionary conservation. While functions of RSPOs are ubiquitous in animal biology, their origin and evolution trajectories are not fully understood. Previous phylogenetic analyses of RSPO homologs in a few vertebrates suggest that the *RSPO* gene family may have originated from the deuterostomate ancestor [56]. The existence of the RSPO protein family has been found in teleost and higher mammals, with most species containing four RSPO members [42,57,58,59]. However, the presence of the *RSPO* gene in other lower animals and its potential subdivision into four subtypes require further in-depth study. To gain a comprehensive understanding of the evolutionary process of the *RSPO* gene family, we conducted a phylogenetic analysis and gene family clustering for the *RSPO* members across 421 animal species. Our focus was on the functional divergence and molecular evolution of the *RSPO* gene family in the context of metazoan evolution. Gene family clustering revealed a new subgroup that does not belong to *RSPO1–4* members. This work broadens our understanding of the evolution of the *RSPO* gene family and provides compelling opportunities for further functional studies on the Wnt signaling pathway. As potential regulators of stem cell proliferation, differentiation, and maintenance, our findings may lead to the discovery of novel RSPO proteins in other species, laying the foundation for regenerative medicine and therapeutic stem cell research.

## 2. Materials and Methods

### 2.1. Genome Data Collection and Processing

Whole-genome assemblies and corresponding GFF annotation files for 430 animals (Supplementary Table S1) were retrieved from NCBI (https://ncbi.nlm.nih.gov/genome/, accessed on 12 March 2024), Ensembl [60], CNCB [61] (https://www.cncb.ac.cn/, accessed on 12 March 2024), and Macgenome (https://www.macgenome.org/, accessed on 12 March 2024), with genome assembly quality assessed using BUSCO [62] (-l mammalia_odb10) and GPA scripts (https://github.com/ypchan/GPA, accessed on 20 March 2024). Multiple sequence alignment of single-copy orthologs was performed using MAFFT v7.525 [63] under --genafpair --maxiterate 1000 parameters for iterative refinement. Alignments were trimmed with trimAl v1.5.rev0 [64] (-gt 0.85-cons 30). Phylogenetic tree reconstruction was conducted using IQ-TREE v2.3.6 [65] (-m JTT+F+R10; -bb 1000), and multiple trees were merged with ASTRAL v5.7.8 [66]. Species phylogenetic trees were imported to the online site Chiplot [67] (https://www.chiplot.online/, accessed on 20 May 2024) for visualization.

### 2.2. Homologs Identification

Two different methods were employed to identify R-Spondins family members. Human RSPO proteins sequences from the HGNC gene families (https://www.genenames.org/, accessed on 20 April 2024) served as queries for BLASTp (v 2.14.1+) [68] searches against 430 proteomes (e-value ≤ 1 × 10^−5^). Then, based on the pfam_scan.py script (https://github.com/aziele/pfam_scan, accessed on 25 April 2024), HMMER-3.3.2 [69] software was used to further predict the domain of the hit protein sequences in the blastp result and to extract those containing both the furin-like cysteine-rich domain (PF15913) and the thrombospondin type 1 domain (PF19028) as the candidate members [70]. We tried to discard some of the sequences and/or pseudogenes by eliminating sequences shorter than 156 amino acids to obtain the final RSPO family members. The sequence length threshold represents 2/3 of the minimum functional human RSPO (RSPO4, 234 aa) [56,71]. Boxplots of species RSPO counts for major vertebrate taxa at the online site Chiplot [67] (https://www.chiplot.online/, accessed on 10 May 2024).

### 2.3. Phylogenetic Analysis and Gene Family Clustering

Validated RSPO sequences were aligned using MAFFT v7.525 [63] with default parameters, followed by gap trimming with trimAl v1.5.rev0 [64] to remove columns containing gaps. Then, IQ-TREE v2.3.6 [65] was employed to construct a maximum likelihood tree with the bootstrap replicates 1000. The ModelFinder [72] was used for the selection of the optimal substitution model. Ggtree v3.12 [73] and ggtreeExtra v1.14 [74] were used to enhance the esthetics of phylogenetic trees. The dgfr v0.0.0.9 [75] (https://github.com/lailaviana/dgfr, accessed on 26 May 2024) was used to determine the optimal number of clusters for grouping members of the *RSPO* gene family (min_clust = 3, max_clust = 8) and calculate the mean/median similarity within each cluster. Finally, combined with the results of gene family clustering and phylogenetic analysis, the grouping of subfamilies was determined. Gene phylogenetic trees with bootstrap values were landscaped and visualized at the online site Chiplot [67] (https://www.chiplot.online/, accessed on 22 July 2024).

### 2.4. Analysis of Structural Diversity of R-Spondins Protein

Based on the pre-trained model esm2_t48_15B_UR50D, ESMfold [76] (https://github.com/facebookresearch/esm, accessed on 17 October 2024) was utilized for the structural prediction of each subfamily protein sequence. US-align [77] was used for the conservative structure alignment of predicted structures across all redefined groups. Three-dimensional model images with conserved motifs were manipulated and rendered in ChimeraX v1.9 [78] software.

## 3. Results

### 3.1. Phylogenetic Analysis and Gene Family Clustering Define the Four Subgroups of the RSPO Family

**Figure 1:**
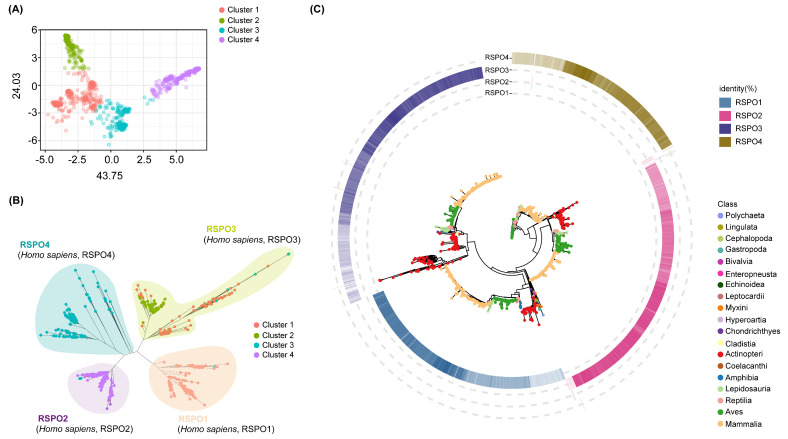
Gene family clustering and phylogenetic analysis. (**A**) Principal Component Analysis (PCA) showing the dispersion pattern of the RSPO gene family. (**B**) Unrooted phylogenetic tree of the RSPO gene family. (**C**) We selected the BLASTP hits for each RSPO family member that had the highest identity to the four human RSPO proteins (RSPO1–RSPO4), and mapped these hits to the periphery of the phylogenetic tree. The four color gradients, ranging from light to dark, represents the degree of similarity from low to high. The gene tree with bootstrap values is shown in Figure S1.

Here, we conducted a comprehensive phylogenetic analysis and gene family clustering to delineate the subgroups within the RSPO family. Homologs of RSPO were identified across 430 species, encompassing major metazoan lineages, including Annelida, Brachiopoda, Mollusca, Arthropoda, Hemichordata, Echinodermata, Actinopteri, Amphibia, Aves, and Mammalia (Supplementary Table S1 and Figure S1). This analysis yielded a total of 1496 RSPO protein sequences, from which a maximum likelihood phylogenetic tree was constructed (Supplementary Table S2 and Figure 1B). Notably, RSPO family members were absent in all analyzed species of Arthropoda, Porifera, Placozoa, Cnidaria, and *Helobdella robusta*. To assess the homology among RSPO family members, we utilized the BLASTp method to compare each identified RSPO candidate sequence to the four human RSPO proteins (hRSPO1-hRSPO4). For each RSPO members, we retained the highest sequence identity value from the four BLASTP alignments and mapped this value to the periphery of the phylogenetic tree (Figure 1C). To estimate and visualize sequence divergence within the RSPO gene families, the R package ‘dgfr’ was applied. A distance matrix was generated by pairwise comparison of each gene sequence within the family, followed by dimensionality reduction. The mean/median similarity and protein sequence counts for each cluster were calculated, determining the optimal number of clusters for RSPO to be four (Figure 1A).

Integrating the optimal number of clusters with the phylogenetic tree topology, the RSPO family was classified into four subgroups (Figure 1A,B). This classification enabled the assignment of human RSPOs to their corresponding subfamilies, adopting vertebrate nomenclature (RSPO1–RSPO4). In the RSPO1 subgroup, orthologs of human RSPO1 were identified in the *Callorhinchus milii* (Chondrichthyes) and *Erpetoichthys calabaricus* (Cladistia), with sequence identities of 62.79% and 60.19%, respectively (Supplementary Table S3). Despite the extensive evolutionary divergence from humans, these species exhibit notable homology. On the contrary, the lowest protein similarity with humans in the RSPO1 group was observed in *Ictalurus punctatus*, a species more closely related to cartilaginous fishes, with an identity of 37.76%. In zebrafish, RSPO1 plays a major role in gonadal development and differentiation, and these expression differences related to sex determination may be conservative in fish but differ from mammalian RSPO1 in other aspects [57]. This observation may explain the lower homology between the Actinopteri and humans in our RSPO1 group (37.76–54.85%). Homologs of RSPO1 were predominantly concentrated in cluster 1, with a few assigned to cluster 3. The RSPO2 and RSPO4 groups were also mainly concentrated in cluster 4 and cluster 3, respectively. The RSPO3 group consists of two main sister clades in the unrooted tree, with clusters 2 and 1 distributed evenly between them, suggesting a special diversity among RSPO3 members. The oldest species containing homolog genes of the RSPO2 and the RSPO3 groups are the same as the RSPO1 group and can be traced back to the Chondrichthyes and Cladistia. The RSPO1, RSPO2, and RSPO3 subfamilies appeared simultaneously in early chordates’ evolution, indicating that the ancestral forms of the RSPO family had diversified prior to the origin of vertebrates.

### 3.2. Four Subgroups Showed Different Evolutionary Patterns

**Figure 2:**
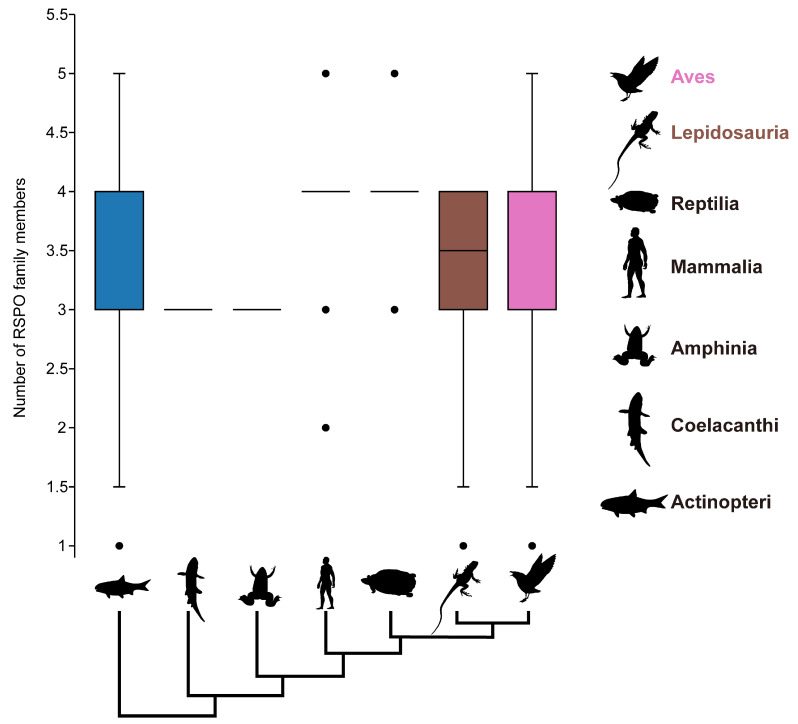
For each vertebrate subclass, the number of RSPO family members is shown as boxplots. A simplified evolutionary tree showing the seven main branches of vertebrates. Different colored boxes are used to distinguish distinct vertebrate subgroups. Boxplots are not shown for Mammalia and Amphibia because the vast majority of the species have a concentration of four copies of RSPO. Coelacanthi and Amphibia, on the other hand, do not have box forms due to the species’ tiny sample size. Figure S2 shows the exact number of four RSPO subtypes that each species possesses. All silhouettes are from PhyloPic (https://www.phylopic.org/, accessed on 10 May 2024).

The analysis of the *RSPO* gene family across 421 species revealed an average of approximately 3.6 members per genome. This extensive dataset enabled us to examine the lineage distribution and evolutionary origins of the *RSPO* gene family in relation to species phylogeny. As depicted in Figure 2, the majority of RSPO members possessed by Mammalia and Amphibia are grouped in four subtypes, although the relative variances in the total number of RSPOs carried by the various species in Actinopteri, Lepidosauria, and Aves are significantly greater. Despite the vast distances in the evolutionary tree, the distribution patterns of RSPO copy numbers in the two lineages, Aves and Actinopteri, are nearly identical. Our findings indicate that the *RSPO* gene family is highly conserved among mammals, with each of the four subtypes (RSPO1–RSPO4) typically represented by a single copy per species (Figure S2). In contrast, protostomes at the base of evolution tree retain only RSPO4, while higher chordates preferentially retain RSPO1 or RSPO2. This observation suggests that the importance of these subtypes varies across different lineage.

The four RSPO subtypes exhibit distinct evolutionary patterns. RSPO1, RSPO2, and RSPO3 members have been lost in protostomes and are unique to the deuterostome lineage (Figure 2). Despite multiple whole-genome duplication events in Chordata, including the two rounds of whole-genome duplication in vertebrates (2R WGD) [79,80] and the teleost-specific 3R WGD [81,82], RSPO1 family members appear to revert to a single-copy state immediately after gene replication. This special conservatism underscores the irreplaceable and critical function of RSPO1 in vertebrates. Following the diversification of the *RSPO* gene family in deuterostomes, the RSPO1 type (the sole RSPO type in deuterostomes) began to repeatedly disappear in the Actinopteri, being replaced by two to three copies of the RSPO3 subtype. As a major vertebrate group, Actinopteri occupy diverse aquatic environments, including extreme habitats such as Antarctica, the deep sea, and sulfide springs. The presence of multiple copies of the RSPO3 subtype may confer adaptive advantages, enabling these species to cope with environmental stresses and reproduce effectively within their ecological niches [83]. The orthologous sequence of RSPO4 is present in both Deuterostomes and Protostomes (e.g., Annelida, Brachiopoda, Mollusca), suggesting that a representative of this subfamily was already present in the last common ancestor of the bilaterians, *Urbilateria*, and gave rise to the subfamily [84]. Current functional research on mammalian RSPO4 is limited, focusing primarily on its differential regulation in nail and bone development [85,86]. While RSPO4 can enhance classical Wnt signaling like the other three RSPOs, its effect is significantly weaker, and its function and efficacy seem to be more restricted in metazoan mammals [87]. Additionally, multiple members of the RSPO4 subfamily have been lost in higher vertebrates, suggesting that the ancestral RSPO family may not have primarily executed the activation function of the Wnt signaling pathway in early animal evolution. Over long periods of evolution and natural selection, this function was gradually retained and strengthened in new sub-functional members. The RSPO4 subtype, representing the most ancient form of the RSPO family, may have retained the other dominant functions of its ancestors, which remain to be explored.

### 3.3. Some RSPO3 Members of Actinopteri Have Three Furin-like Repeats

Our analysis revealed that certain Actinopteri species possess multiple copies (two or three) of the RSPO3 gene, while other vertebrate lineages retain a single copy. To investigate this divergence, we further compared the protein sequences of RSPO3 across various species and inferred their phylogenetic relationships. We rooted the maximum likelihood tree with the RSPO3 protein sequence from *C. milii*, a representative cartilaginous fish species that diverged from the remaining gnathostome (jawed vertebrate) lineage approximately 450 million years ago [88]. The phylogenetic analysis delineated two distinct evolutionary branches, one comprising Actinopteri and the other encompassing higher vertebrates (Lepidosauria, Reptilia, Aves, and Mammalia). These branches are sister groups, with their common ancestor closely related to the RSPO3 of Amphibia. Our results show that in the Actinopteri lineage, the *RSPO3* gene has significantly diverged from that of other vertebrates.

**Figure 3:**
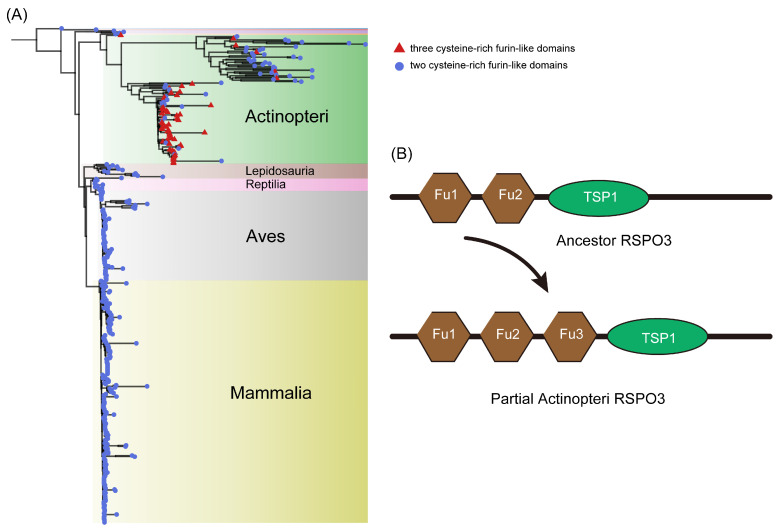
Phylogenetic tree and domain structure analysis of RSPO3 across vertebrate lineages. (**A**) Phylogenetic tree of RSPO3 protein sequences from various vertebrate species, including Actinopteri, Lepidosauria, Reptilia, Aves, and Mammalia. The tree is rooted using the RSPO3 sequence from *C. milii*. The species are color-coded by taxonomic group, with Actinopteri in green, Lepidosauria in pink, Aves in light yellow, and Mammalia in dark yellow. Red and blue triangles indicate species with distinct RSPO3 domain variations. (**B**) Schematic representation of the domain structure of RSPO3 proteins. The ancestral RSPO3, found in most vertebrates, contains two conserved cysteine-rich furin-like (Fu) domains and one thrombospondin type 1 (TSP1) domain. In some Actinopteri species, RSPO3 homologs feature an additional third Fu domain, resulting in partial amplification of the RSPO3 structure, which is shown as “Partial Actinopteri RSPO3”.

RSPO family members are characterized by three conserved functional domains: two repeated cysteine-rich furin-like domains and one TSP1 domain. Our structural domain analysis confirmed the presence of these domains in all higher vertebrate RSPO3 branches, as well as in the RSPO3 subtypes of early chordate species (*C. milii* and *Latimeria chalumnae*) (Figure 3). However, in some Actinopteri species, the number of FU domains in the RSPO3 isoform varied. Notably, we identified RSPO3 homologs with an additional third FU domain in certain Actinopteri species, a feature not observed in RSPO3 members of other lineages (Supplementary Table S4). This finding aligns with previous phylogenetic analyses of RSPO3 in Cynoglossus semilaevis and a limited number of vertebrates. The variation in FU domain number among Actinopteri species may be due to independent amplification events or retention of ancestral states in different branches.

Whole-genome duplication (WGD) events are often associated with evolutionary innovation and provide additional genetic material for species adaptation [89,90]. Actinopteri experienced multiple WGD events, including two rounds at the vertebrate lineage roots (VGD1 and VGD2) and a third round (TGD) 320–350 million years ago (Mya) [82,91]. Subsequent specific WGDs have been described in the teleost lineage [92,93]. Our phylogenetic analysis of RSPO3 homologous proteins in Actinopteri revealed a clear evolutionary pattern. *E. calabaricus*, an early-diverged teleost, exhibited RSPO3 forming an independent branch with two FU domains at the N terminal, consistent with most vertebrates. In contrast, *Lepisosteus oculatus* displayed RSPO3 as a separate branch with an amplified FU domain, indicating early FU domain amplification. In the remaining teleost branches, RSPO3 subtypes with two or three FU domains were present, suggesting multiple independent amplification events or retention of ancestral states. We hypothesize that the ancestral RSPO3 gene possessed two FU domains. Subsequent specific WGDs in Actinopteri led to the fixation of RSPO3 family members with multiple FU domains in certain species, contributing to adaptive evolution in diverse environments (Figure 3B).

### 3.4. The Identification of Conservative Motif

**Figure 4:**
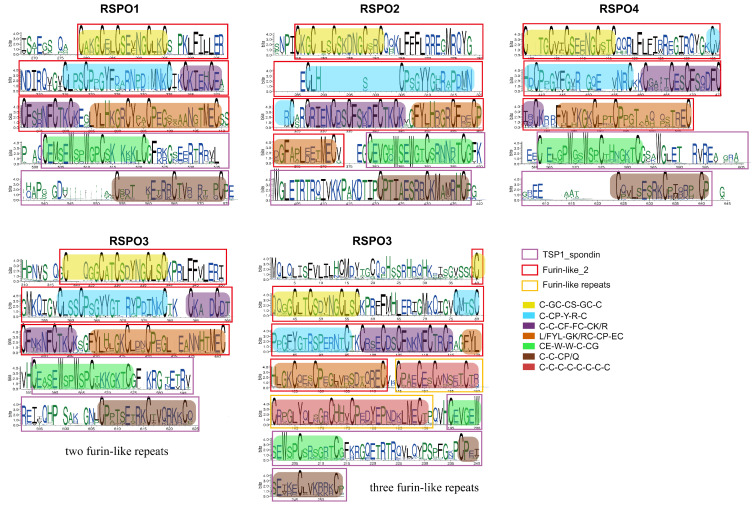
Conserved features of *RSPO* gene family during animal evolution. The Seqlogo diagram displays the conserved sequence features of four RSPO subgroups that have evolved in more than 400 known animal species. Different color bars are overlaid to highlight them.

SeqLogo analysis of homologous protein sequences spanning more than 400 animal species was employed to resolve conserved structural motifs with high fidelity (Figure 4). Comparative sequence analysis of RSPO homologs revealed two evolutionarily conserved cysteine-rich motifs within the N-terminal furin-like_2 domain (Pfam: PF15913.10). These motifs, designated Motif 1 (C-GC-CS-GC-C-C-CP-Y-R-C) and Motif 2 (C-C-CF-FC-CK/R-FYL-GK/RC-CP-EC) near the N-terminal position, where “-” denotes variable-length spacers, were universally present in all RSPO subtypes, including divergent RSPO3 paralogs. Structural annotation of PF15913.10 confirmed that these motifs constitute tandem cysteine-rich furin-like repeats critical for domain architecture. In contrast, the C-terminal region exhibited a highly conserved TSP1_spondin domain (Pfam: PF19028.5), defined by a signature motif Motif 3 (CE-W-W-C-CG-C-C-CP/Q), which distinguishes the RSPO family. Notably, RSPO3 members uniquely harbored an additional intermediate segment containing eight conserved cysteines, forming a third furin-like repeat absent in other subtypes. Sequence alignment demonstrated that this tertiary repeat shares higher similarity with Motif 2 (secondary repeat) than with Motif 1, suggesting lineage-specific duplication and divergence.

All RSPO proteins displayed strict conservation of cysteine residues at both termini, consistent with their role in stabilizing tertiary structures via disulfide bonds. However, RSPO1 and RSPO4 groups exhibited reduced conservation, retaining only six core cysteines. Phylogenetic analysis highlighted RSPO4 as the most divergent subtype with sequence variability spanning cartilaginous fishes to mammals, potentially reflecting its ancestral origin. Post-translational modification analysis identified a conserved WXXW motif within the PF19028.5 domain, a hallmark of C-mannosylation, where α-mannosyl groups are enzymatically linked to tryptophan residues [94].

The binding of RSPO to two receptors has emerged as a key regulatory mechanism for activating Wnt signaling. In the FU-CR1 subdomain, Arg66 and Gln71, were essential for high-affinity binding to ZNRF3/RNF43, as alanine substitutions (R66A or Q71A) abolished this interaction. Similarly, FU-CR2 residue Phe106 and Phe110 were indispensable for LGR4 binding, while F106A/F110A mutants disrupted complex formation [95]. Clinically, pathogenic mutations in RSPO4 (R65W, Q70R, and G72R) linked to hereditary nail disease [96,97,98] are mapped to homologous positions in other RSPO subtypes, underscoring the functional necessity of these conserved residues (Figure 4).

### 3.5. The Analysis of Spatial Structure of RSPO Protein

Structural and functional conservation analyses of RSPO proteins revealed distinct evolutionary and functional features across subgroups. To explore structural diversity and conservation, an ESMfold-predicted three-dimensional model of subgroup-specific RSPO sequences was aligned using US-align, with TM-values quantifying structural similarity. Non-conserved regions were rendered partially transparent in visualization to emphasize conserved motifs, which were color-coded for clarity. Structural alignment demonstrated that all RSPO subgroups exhibit highly conserved spatial conformations in their FU domains and TSP domains. Notably, the “-CE-W-W-C-CG-C-C-CP/Q-” motif within the TSP domain adopted a spatial compact organization in RSPO2 and RSPO3 subgroups, which possess three FU domains (3FU architecture). In contrast, this motif exhibits a more dispersed spatial arrangement in other subgroups. These observations suggest that while the core motif composition is universally conserved, non-motif residues in the TSP domains of non-RSPO2/3 subgroups exhibit greater structural plasticity, suggesting functional diversification.

**Figure 5:**
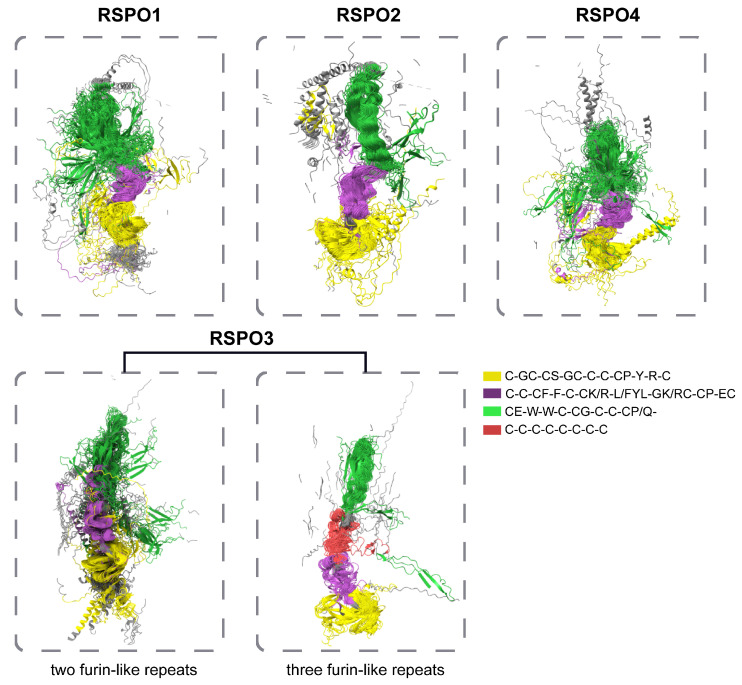
Comparison of the spatial structure of different groups of RSPO proteins. The comparison results of spatial structure of proteins in each subgroup of RSPOs show that the non-conserved spatial structure region is set to 100% transparency, and the corresponding conserved motifs in Figure 4 are distinguished by different colors.

Comparative analysis highlighted unique features of the RSPO1 subgroup, including a significantly extended N-terminal conserved region (highlighted in gray in visualizations). Given prior evidence implicating the RSPO N-terminus in secretory regulation [14,30], this pronounced conservation implies enhanced secretory efficiency in RSPO1, potentially reflecting its critical role in extracellular signaling and developmental processes. In contrast, other subgroups likely rely on N-terminal FU domain repeats for secretion. Phylogenetic distribution analysis further revealed that RSPO4, present exclusively in basal metazoans, retains an evolutionarily ancient C-terminal conserved region distinct from the TSP domain. While RSPO2 homologs in Deuterostomia possess an extended C-terminal region, the shorter yet conserved C-terminus in RSPO4 suggests progressive evolutionary refinement of this domain to augment functional specificity in derived lineages. Subgroup classification based on FU domain organization identified two structural categories, one containing the traditional two repeated FU domains, and the other including a derived 3FU architecture featuring an additional FU domain between FU2 and the TSP domain (Figure 5). Structural and sequence conservation analyses revealed that the third FU domain in 3FU subgroups (RSPO2/3) shares high homology with the canonical FU2 domain. Previous studies have shown that in human RSPO1, the FU1 and FU2 mediate binding to ZNRF3 and LGR4, respectively [99], while the 3FU structure of RSPO3 correlates with enhanced Wnt signaling inhibition in zebrafish embryogenesis [59]. These observations suggest that FU domain duplication may confer expanded interaction interfaces or altered receptor binding dynamics in specific lineages.

## 4. Discussion

The WNT signaling pathway, a highly conserved mechanism integral to embryonic development, tissue regeneration, and stem cell maintenance, underscores its profound biological significance across Metazoa. The regulation of the canonical Wnt/β-catenin signaling pathway by RSPO was fundamental to the development of multicellular organisms over evolution. In bone, RSPO1 was initially reported to promote and enhance osteoblast differentiation of mouse C2C12 cells through a synergistic effect with Wnt3a [32]. RSPO2 is essential for animal embryo and limb development [32,46]. RSPO3 was shown to be essential for cardiac development [100]. Mutations in the RSPO4 can underlie inherited anonychia/hyponychia [85]. Genetic research on humans and mice has shown that *Rspos* has various biological functions in vivo. While previous studies have elucidated the evolutionary trajectories of the WNT ligand family and its antagonists (e.g., Dkk proteins), the origins and diversification of the R-spondin (RSPO) family—a key agonist of WNT signaling—remain poorly resolved. To address this gap, we conducted a comprehensive phylogenetic and genomic survey of RSPO homologs across 430 animal species, identifying 1496 RSPO family members. Notably, no RSPO homologs were detected in basal metazoan lineages, including annelids and cnidarians, suggesting an emergence coinciding with bilaterian evolution. Phylogenetic reconstruction and gene family clustering robustly delineated the RSPO family into four evolutionary conserved types (RSPO1–RSPO4) (Figure 1). This classification aligns with prior reports of sister-group relationships between RSPO1/RSPO3 and RSPO2/RSPO4, respectively [101].

Subtype distribution analyses revealed dynamic copy number variation across taxa. In agreement with Clevers et al., no RSPO homologs were identified in the model species Drosophila [56]. In general, copy numbers tended to increase from lower animals with simple physiological structures to higher animals with complex metabolism (Supplementary Table S4). We hypothesized that ancestral forms of the RSPO family diverged sometime before vertebrate origin. Then, four subtypes of the RSPO family showed different evolutionary patterns. In particular, the RSPO3 exhibited lineage-specific expansions in teleost and was likely driven by whole-genome duplication (WGD) events and adaptive diversification. In contrast, RSPO1 retained strict single-copy conservation in vertebrates, underscoring its non-redundant functional role. Strikingly, RSPO4 homologs were identified in both Deuterostomata and Protostomata (Annelida, Brachiopods, Molluscs), suggesting an ancestral origin predating the divergence of Bilateria. This phylogenomic pattern positions RSPO4 as the most ancient isoform, originating in the last common bilaterian ancestor, *Urbilatera* [84]. In summary, our analysis reveals a trend from a single subtype and single copy in protostomes to four subtypes and multiple copies in vertebrates, reflecting the adaptive evolution of the *RSPO* gene family across animal groups. There are also several studies suggesting that innovations in gene and protein regulation in proto-hostile animals may also have contributed to animal evolution [102,103,104]. Therefore, we also consider an opposite scenario whereby the enrichment of RSPO family member types and the increase in copy number may lead to the adaptive evolution of animals and thus the generation of new animal populations, rather than being merely the result of adaptive evolution of animals.

Structural analyses revealed divergent evolutionary constraints among RSPO family members. While all RSPO proteins retain conserved N-terminal signal peptides and furin-like (FU) domains critical for ligand–receptor interactions, teleost RSPO3 uniquely encodes a third FU repeat, a synapomorphic feature absent in other vertebrate lineages. However, comparative genomics clarified that this tri-FU architecture is not ubiquitous among teleosts, as several species retain the ancestral bi-FU structure observed in tetrapods and chondrichthyans. Sequence conservation mapping (SeqLogo; Figure 4) further highlighted stringent purifying selection on residues mediating molecular interactions: arginine (R66) and glutamine (Q71) within the FU-CR1 domain (essential for ZNRF3/RNF43 binding) and phenylalanine residues (F106/F110) in FU-CR2 (required for LGR4 engagement) [95]. These motifs exhibit near-universal conservation across more than 400 animal species, emphasizing their functional indispensability and potential as therapeutic targets. Three-dimensional structural predictions corroborated high spatial conservation of FU and TSP domains across homologs, with RSPO2/3 subtypes displaying particularly rigid TSP domain conformations. RSPO1 uniquely harbors an N-terminal conserved region implicated in the secretion regulation, while RSPO4 possesses a C-terminal extension adjacent to its TSP domain, a structural innovation potentially linked to functional specialization. Intriguingly, members with tri-FU repeats (e.g., teleost RSPO3) exhibited altered domain architectures, suggesting neofunctionalization in receptor binding affinity or signaling modulation.

Reports have described that huge disturbances in multicellular layer functionalities can lead to cancer [105,106]. An intact and functionally normal Wnt pathway is one that belongs to the multicellular layer function, and disruption (especially overexpression) of RSPO members as well-established positive modulators of the Wnt pathway implies a severe disturbance of the multicellular layer function, which may ultimately lead to tumorigenesis [37,38,41]. Cancer, as a difficult problem in the history of human diseases, has not been completely overcome, and the common treatments at present are radiotherapy and surgical resection [107,108]. But these methods will only bring short-lived results before the cancer cells develop resistance to the drugs and cause more intense side effects from attacking normal cells. According to therapeutic strategies for cancer regression, cell proliferation is the most entrenched capacity that any cell (including cancer cells) possesses after 4 billion years of evolution, and it is also an advantage for cancer cells; however, the emergence of regulation of cell proliferation is significantly later than cell proliferation itself. Many layers of regulation of cell proliferation are much more recent than the evolution of cell proliferation itself (in the last billion years), and this is seen as a weakness of cancer cells [109]. Most current cancer medicines target this dominant cell proliferation approach. The inhibition of *RSPO* overexpression may present a promising therapeutic strategy, aligning with the principle of ‘Targeting cancer’s vulnerabilities rather than its strengths’ [110]. For example, a *RSPO4* mutant (Q65R) was identified that retains strong LGR binding but no longer activates Wnt signaling. Drug conjugates based on this mutant efficiently reduced tumor growth without causing intestinal enlargement or other side effects [111]. Therefore, our structural investigation of RSPO family members are extremely important, as they open up new avenues for the development of RSPO-targeted cancer treatments. Overall, these findings establish a robust evolutionary framework for the RSPO family, resolving its emergence in early bilaterians and subsequent diversification through WGD, domain duplication, and lineage-specific selection. The conserved molecular architecture of RSPO members, coupled with their dynamic structural evolution, provides mechanistic insights into their functional diversification across Wnt-mediated processes. Future studies dissecting isoform-specific interactions with LGR receptors and ZNRF3/RNF43 E3 ligases will be critical for understanding their context-dependent roles in development, homeostasis, and disease.

## 5. Conclusions

This study represents the first comprehensive investigation of the evolutionary trajectory and diversification of the *RSPO* gene family across diverse metazoan lineages, including nearly all major vertebrate clades. By integrating phylogenetic reconstruction, copy number variation analyses, conserved amino acid residue alignments, and comparative structural modeling, we elucidated the origin and functional divergence of the RSPO paralogs. Specifically, our findings systematically reclassify the four RSPO subtypes (RSPO1–RSPO4) across the animal kingdom, extending the established vertebrate nomenclature to ancestral bilaterian lineages, *Urbilateria*. Crucially, RSPO4 emerges as the ancestral subtype originating in the last common ancestor of bilaterian animals (*Urbilateria*), predating the emergence of vertebrates. Furthermore, we uncovered distinct evolutionary trajectories and lineage-specific domain amplifications characterizing the RSPO3 subclass, suggesting unique functional constraints driving its diversification. Comparative analysis of conserved residues, structural motifs, and tertiary conformations across non-modal species revealed evolutionarily constrained regions that may serve as potential ligand-binding or signaling interfaces. These structural insights provide a framework for identifying therapeutic targets in oncology, particularly for cancers driven by RSPO-mediated Wnt signaling dysregulation. Additionally, our systematic reclassification of RSPO orthologs in proto-oral and posterior animal lineages resolves longstanding ambiguities in gene family nomenclature while offering critical insights into functional innovation across evolutionary timescales.

## Data Availability

The original contributions presented in the study are included in the Supplementary Material; further inquiries can be directed to the corresponding authors.

## Supplementary Materials

The following supporting information can be downloaded at: https://www.mdpi.com/article/10.3390/genes16050477/s1, Figure S1: Maximum likelihood (ML) phylogenetic tree of 1496 RSPO gene family members with bootstrap values for each branch; Figure S2: The distribution of copy number of four subtype members among the RSPO gene family in animals; Table S1: The classification and genomic data source of 430 animals; Table S2: The basic statistics of the RSPO protein family members; Table S3: The Blastp identity statistics of RSPO gene family members; Table S4: The NCBI-CDD domain identification results of RSPO3 subfamily members.

## Abbreviations

The following abbreviations are used in this manuscript:

RSPO	the R-spondin family
TSP domain	Thrombospondin Type 1 Repeat domain
FU domain	Repeated cysteine-rich furin-like domain
